# The Patients' Column

**Published:** 1914-06-13

**Authors:** 


### The Patients' Column.

Note.—Contributions to this series are invited. Previous articles appeared on April 18, 25, May 2 and 9.

### V.—A SPECIAL AND A DISTRICT HOSPITAL EXPERIENCE.

I had the misfortune during the hot summer of 1911 to require immediate operation on two occasions—first, for ovarian tumours, and next, acute appendicitis. For the former I was taken to a special hospital as an ordinary patient. Our means were very limited, and a nursing home was impossible. Well do I remember how strange and lonely and sad I felt when I entered the hospital and a nurse took charge of me! After asking a few questions, she led me to a bathroom and asked me to take a bath and prepare for bed. The bath was welcome and refreshing after the heat and dust of a railway journey. I fully recognised how needful a bath is in many cases, and to resent would have been absurd. I was then conducted to a fine, large, airy ward, made gay with flowers. All the beds were occupied except mine, and I made number nine. During my stay of five weeks I found no cause of complaint. Doctors and nurses were always attentive and sympathetic, and no favour shown.

Our dear little night nurse was a jewel! All night long bells were ringing, first in one ward, then in another. Softly and swiftly she flew, never a moment's rest and never a grumble, always ready with a kind word and gentle touch. For the first week after operation we were called "babies." Babies indeed we were; lying on our backs, all trussed up, we were perfectly helpless.

Our day began about five; it did seem early, but when one knew all the night nurse had to do not one bit too early. We breakfasted at 6 a.m. on tea and bread and butter. If the doctor allowed us eggs we could have them sent in, and nurse boiled them. The other meals were plain and good, and always enough.

About 7.30 the day nurses trooped in; however they felt, they were always fresh, bright, and full of spirits, and I am sure no domestic servant ever worked harder than they. On the whole, there were few grumblers. One patient was very trying; nothing pleased her. She was constantly regretting the glass of stout she was accustomed to take with her dinner.

I left the hospital with feelings of heartfelt gratitude. A month later, more dead than alive, I was taken in an ambulance to the district hospital for acute appendicitis. An operation took place at midnight on a Sunday. Here the rules and regulations were less rigorous than at the special hospital, but the treatment was splendid. To the skill of the doctors, matron, sisters, and nurses I feel I owe my life, and to them I must always owe a deep debt of gratitude. This little hospital was like home, and often I blessed those whose generosity had made it possible.

